# Therapeutic efficacy and mechanism of water-soluble extracts of Banxiaxiexin decoction on BALB/c mice with oxazolone-induced colitis

**DOI:** 10.3892/etm.2014.1890

**Published:** 2014-08-08

**Authors:** XUEWEI WANG, JINGHUI YANG, QIN CAO, JIANMIN TANG

**Affiliations:** 1 Department of Gastroenterology, Putuo Hospital, Shanghai University of Traditional Chinese Medicine, Shanghai 200062, P.R. China; 2 Department of Pathology, Putuo Hospital, Shanghai University of Traditional Chinese Medicine, Shanghai 200062, P.R. China

**Keywords:** Banxiaxiexin decoction, oxazolone, ulcerative colitis, interleukin-5, interleukin-13

## Abstract

The aim of the present study was to investigate the therapeutic effects of water-soluble extracts of Banxiaxiexin decoction, a classical traditional Chinese medicine formulation, on BALB/c mice with experimentally induced ulcerative colitis (UC). Water-soluble extracts of Banxiaxiexin decoction were intragastrically administered to BALB/c mice with oxazolone (OXA)-induced colitis. Sulfasalazine (SASP) was administered intragastrically to OXA-treated mice to establish the SASP group (positive control). Following drug administration, the disease activity index (DAI) and the histopathological inflammation score were recorded. In addition, the expression levels of interleukin (IL)-5 and IL-13 mRNA in the colonic tissue were determined by fluorescent quantitative polymerase chain reaction. The DAI and histopathological inflammation score of the model group were significantly greater compared with those of the control group, and the mRNA expression levels of IL-5 and IL-13 in the colonic tissue were also significantly higher in the model group compared with those in the control group. The intragastric administration of water-soluble extracts of Banxiaxiexin decoction significantly lowered the DAI and histopathological inflammation score. The mRNA expression levels of IL-5 and IL-13 in the colonic tissue were also significantly lowered. The therapeutic effect of Banxiaxiexin decoction was found to be comparable to that of SASP. In conclusion, the results from the present study demonstrate that water-soluble extracts of the traditional Chinese medicine formulation Banxiaxiexin decoction have a therapeutic effect on BALB/c mice with OXA-induced colitis.

## Introduction

Inflammatory bowel diseases (IBDs), which include ulcerative colitis (UC) and Crohn’s disease (CD), are chronic inflammatory disorders of the gastrointestinal tract. Currently, a number of drugs, including 5-aminosalicylic acid (5-ASA) drugs, for example sulfasalazine (SASP) and mesalazine, are used for the treatment of UC (1). In addition, corticosteroids, azathioprine and mercaptopurine are also used for treatment of UC (2). However, these drugs are not the optimal choice for long-term treatment and are used only if patients do not achieve remission with 5-ASA (3). Furthermore, these drugs have significant risk factors, including an increased risk of cancer, tuberculosis and heart failure (4). Therefore, the development of a novel strategy for the treatment of UC is of importance.

Notably, ~21% of patients with IBD use alternative treatments (5). Complementary and alternative medicine (CAM) is becoming increasingly popular and is used to treat patients with UC in China. CAM is considered as an effective adjunct treatment by physicians and patients. Banxiaxiexin decoction, containing seven commonly used plants (*Pinellia ternata*, *Scutellaria baicalensis*, ginseng, Rhizoma Coptidis, liquorice, ginger and red jujube), is a classical Chinese medicine formulation. It has been widely used for centuries to treat IBD in clinical practice in China (6). This traditional Chinese medicine formulation is a promising agent for the treatment of many chronic gastrointestinal diseases, including chronic gastritis, peptic ulcer disease and certain chronic intestinal diseases. However, the use of Banxiaxiexin decoction for the treatment of UC has yet to be reported, and the pharmacological mechanism is not yet clearly defined. Further studies are required prior to it being recommended for treatment of UC.

Therefore, the aim of the present study was to investigate the efficacy of Banxiaxiexin decoction for the treatment of UC. To analyze the therapeutic effects of novel drugs, an appropriate animal model is necessary. A modified experimental model of UC in BALB/c mice was established in the present study. A low dose of the haptenating agent oxazolone (OXA) was used to induce colitis. OXA promotes the production of Th-2 type cytokines, resulting in lesions characterized by lymphocyte infiltration.

## Materials and methods

### Animals

The study protocol was approved by the ethics committee of Shanghai University of Traditional Chinese Medicine (Shanghai, China) and performed in accordance with the Guide for the Care and Use of Laboratory Animals (7). Male BALB/c mice were obtained from the Shanghai Laboratory Animal Center (Shanghai, China). Specific-pathogen-free male BALB/c mice, weighing 18–20 g, were used for the OXA-induced colitis model. Mice were housed in polycarbonate cages and fed a standard chow and tap water *ad libitum*.

### Induction of colitis

Colitis was induced as previously described, with modifications (8). Briefly, a 2x2 cm field of the abdominal skin was shaved, and 200 μl 3% OXA (Sigma, St. Louis, MO, USA) in 100% ethanol was applied to pre-sensitize the BALB/c mice, and this was repeated the next day. Then, each BALB/c mouse was slightly anesthetized with 0.2% pentobarbital intraperitoneally. A 3.5-F polyurethane catheter was inserted 4 cm into the lumen of the colon, via the anus. A solution of OXA (150 μl; 1% OXA dissolved in 50% ethanol) was administered into the colon via the catheter. Following injection of the OXA solution, the catheter was removed and the mouse was held vertically for 60 sec. For the control group, the same amount of ethanol was injected, instead of OXA solution.

### Preparation of water-soluble extracts of Banxiaxiexin decoction and intragastric administration of SASP and the water-soluble extracts

The Banxiaxiexin decoction consists of *Pinellia ternata* 12 g, *Scutellaria baicalensis* 6 g, ginseng 9 g, Rhizoma Coptidis 3 g, liquorice 9 g, ginger 9 g and four red jujube fruits. To obtain the water-soluble extracts, a volume of water 10-fold greater than that of the decoction was added, and the resulting mixture was heated for 1 h. This was repeated three times. Then, all the extraction liquids were collected, dried and stored at 4°C.

Extracts of the decoction were administered intragastrically (10 mg/g bodyweight) to the OXA-treated BALB/c mice to establish the Banxia group. SASP intragastric administration (20 mg/g bodyweight) was used to treat the OXA-treated mice in the SASP group, which acted as a positive control. Even though there was no specific interval between administration of OXA and decoction/SASP treatment, the mice were held vertically for 60 sec. Intragastric administration of the decoction extract or SASP was performed once a day for 7 days. In the model group, an equal volume of water was administered to the OXA-treated mice.

### Disease activity index (DAI) analysis

The DAI was determined at the end of the treatment period, according to the parameters outlined in Table I.

### Histological analysis

The mice were sacrificed at end of the treatment period. Colonic tissues were dissected and washed with Hank’s balanced salt solution (containing 10 μg/ml gentamicin, 100 U/ml penicillin and 100 μg/ml streptomycin). The tissues were then fixed in 10% natural buffered formalin, embedded in paraffin, cut into tissue sections (5 μm thick) and stained with hematoxylin and eosin (H&E). The stained sections were examined for evidence of colitis using the Wirtz’s criteria (9) (Table II).

### Analyses of mRNA expression levels of interleukin (IL)-5 and IL-13 by quantitative polymerase chain reaction (qPCR)

Total RNA was extracted from the colon after the 7 days of therapy (after colitis induction) using the TRIzol^®^ method (TRIzol reagent; Invitrogen Life Technologies, Carlsbad, CA, USA) (10). Reverse transcription was performed using a cDNA Synthesis kit (Sangon Biotech, Shanghai, China), in accordance with the manufacturer’s instructions. qPCR was performed in a 10 μl final volume containing the following: 5 μl 2X SYBR Green I master mix (Qiagen, Hilden, Germany); 1 μl 5 μM forward primer and 1 μl 5 μM reverse primer (IL-5 primers: forward, 5′-AAGGATGCTTCTGCACTTGA-3′ and reverse, 3′-GGAAGCCTCATCGTCTCATT-5′, IL-13 primers: forward, 5′-AGCATGGTATGGAGTGTGGA-3′ and reverse, 3′-TTGCAATTGGAGATGTTGGT-5′); and 3 μl diluted cDNA. Following an initial denaturation step at 50°C for 2 min and 95°C for 10 min, temperature cycling was initiated. Each cycle consisted of denaturation at 95°C for 15 sec, annealing at 60°C for 1 min and elongation at 72°C for 20 sec. In total, 40 cycles were performed. Mouse β-actin was used as the control for normalizing the quantities of transcripts of IL-5 and IL-13.

### Statistical analysis

The SNK-q test was used to compare the distributions of the groups. P<0.05 was considered to indicate a statistically significant difference. Data were analyzed using SPSS software, version 13.0 (SPSS, Inc., Chicago, IL, USA).

## Results

### DAI score

**Figure 1:**
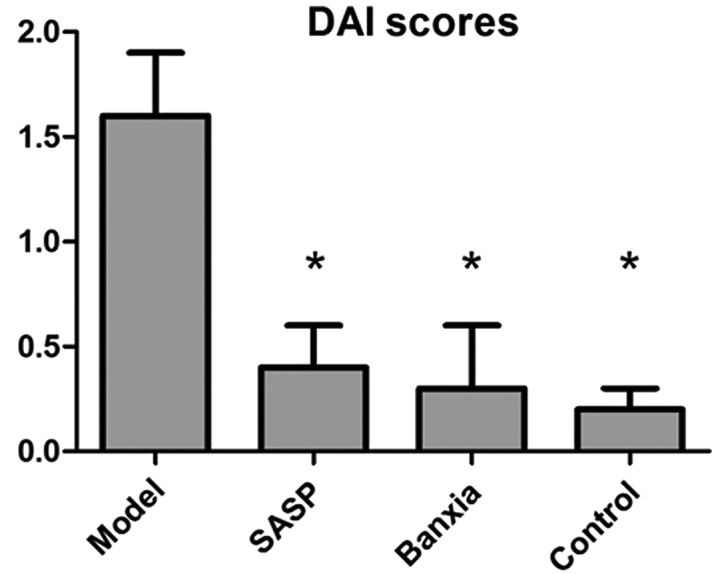
DAI scores for various groups. The DAI scores were measured after 7 days of treatment. Treatment with SASP or Banxiaxiexin decoction reduced the DAI score, and a significant difference was observed compared with the model group. ^*^P<0.05 vs. the model group (SNK-q test) Each column represents the mean ± standard deviation. Control, vehicle control group; Model, mice with colitis; SASP, mice with colitis treated with sulfasalazine; Banxia, mice with colitis treated with Banxiaxiexin decoction.

The appearance of the fur, vitality, weight, appetite and stools in the control group were normal. Yellow fur, lethargy, depression, loss of appetite, significant weight loss, diarrhea and bloody stools were observed in the OXA-induced mice. The condition of the mice following treatment with SASP or Banxiaxiexin decoction was significantly better than that of the mice in the model group. The DAI scores for the mice in those groups are shown in Fig. 1. The DAI score of the model group was significantly higher compared with that of the control group (P<0.05). The DAI scores of the SASP and Banxia groups were significantly lower compared with that of the model group (P<0.05); however, no significant difference between the SASP and Banxia groups was observed.

### Histological examination of inflammation

**Figure 2:**
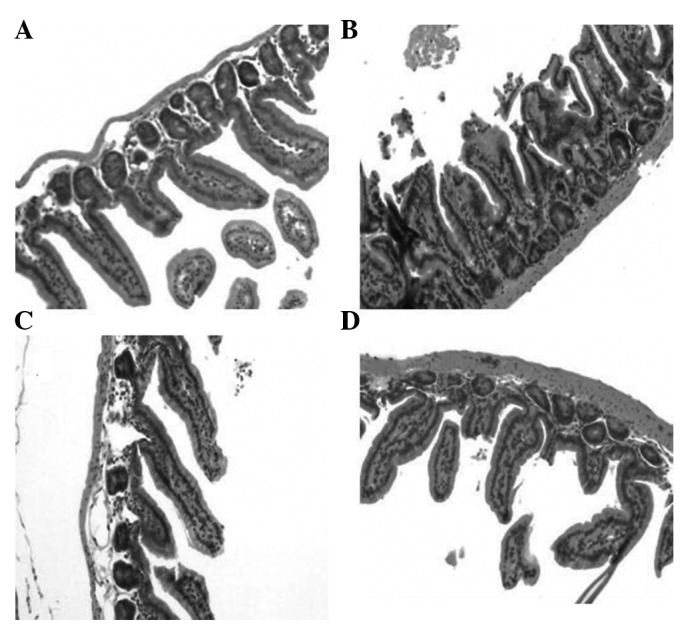
Representative images from the hematoxylin and eosin-stained sections of the colon from the different groups of mice. (A) control group, (B) model group (oxazolone-induced), (C) SASP group (oxazolone-induced and SASP-treated) and (D) Banxia group (oxazolone-induced and Banxiaxiexin decoction-treated). SASP, sulfasalazine.

Histological examination of colonic sections from the OXA-treated mice revealed mucosal inflammation, characterized by the presence of mononuclear cell infiltration, primarily lymphocytes, monocytes and plasma cells, but also neutrophils and eosinophils, a reduction of goblet cells, increased vascular density and a thickening of the colonic wall. These lesions were reduced in severity in the SASP and Banxia groups (Fig. 2). Scores for the inflammation-associated histological changes in the colon for the model, SASP, Banxia and control groups are shown in Table III. The score was significantly increased in the model group compared with that in the control group. Furthermore, scores were decreased in the SASP and Banxia groups compared with that in the model group. However, no significant difference was identified between the SASP and Banxia groups.

### mRNA expression of IL-5 and IL-13

**Figure 3:**
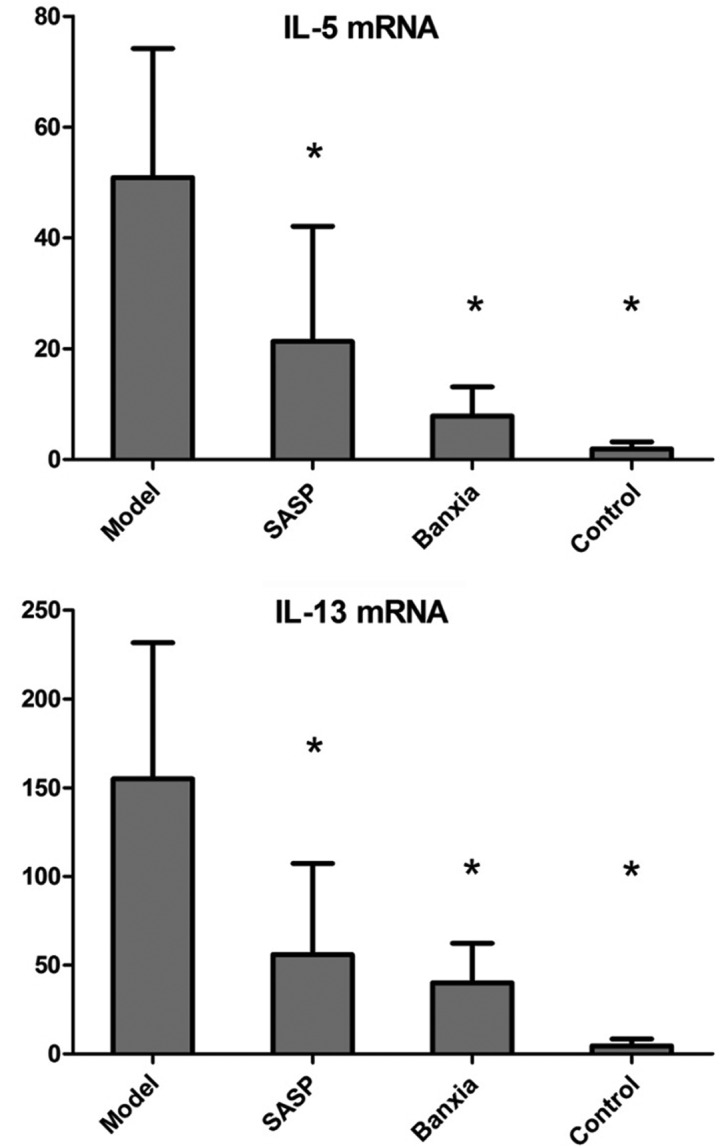
Changes in the mRNA expression levels of IL-5 and IL-13. Colitis was induced via intrarectal administration of OXA solution following skin sensitization, and total RNA was extracted after 7 days of treatment. Cytokine gene expression levels were determined using fluorescent quantitative polymerase chain reaction. The optical density of each band was normalized against the β-actin expression level and calculated as relative signal intensity. Each column represents the mean of 10 mice. ^*^P<0.05, vs. model group (SNK-q test). Each column represents the mean ± standard deviation. IL, interleukin; OXA, oxazolone; Control, vehicle control; Model, mice with colitis; SASP, mice with colitis treated with sulfasalazine; Banxia, mice with colitis treated with Banxiaxiexin decoction.

The expression levels of IL-5 mRNA were significantly increased in the model group compared with those in the control group; however, the expression levels were decreased in the SASP and Banxia groups, compared with those in the model group. Similar to IL-5, the expression levels of IL-13 mRNA were also significantly increased in the mice treated with OXA compared with those in the control group. OXA-treated mice that received SASP or Banxiaxiexin decoction exhibited significant reductions in the expression level of IL-13 mRNA. However, no significant difference was identified in the expression levels of IL-5 and IL-13 mRNA between the SASP- and Banxia decoction-treated mice. The expression levels of IL-5 and IL-13 mRNA for the different groups are shown in Fig. 3.

## Discussion

In the present study, the therapeutic effects of the water-soluble extracts from the traditional Chinese medicine formulation Banxiaxiexin decoction on UC in an animal model was investigated. UC was induced in BALB/c mice by the intrarectal administration of a low dose of OXA, following skin sensitization with OXA. Morphological changes, analyzed using histological methods, were similar to the tissue damage observed in patients with UC. The morphological changes were characterized by a thickening of the colonic wall, depletion of goblet cells, increased vascular density and lymphocyte infiltration.

SASP, a 5-ASA drug used for the treatment of IBD, and the water-soluble extracts from Banxiaxiexin decoction, were used in the present study for the treatment of UC. It was found that Banxiaxiexin decoction exerted a significant anti-inflammatory effect and the therapeutic effect of the Banxiaxiexin decoction was found to be similar to that of SASP. BALB/c mice treated with Banxiaxiexin decoction showed improvements in body weight and the appearance of feces, and alleviation of bloody stools. Furthermore, the DAI score, an indicator of the severity of intestinal inflammation, was increased in the model group, but decreased in the SASP and Banxia treatment groups.

The mRNA expression levels of cytokines from mononuclear cells, primarily CD4^+^ T-helper-2 (Th-2) cells and NK-T cells, were determined using qPCR. Increased IL-5 and IL-13 mRNA expression levels were observed in the model group. A previous study suggested that disruption of the intestinal mucosal immune system is involved in the pathogenesis of IBD (11). Several pathways, including the Th-1 and Th-2 immune response pathways, are considered to have a role in development of IBD. It should be noted that the Th-1 immune response may have an important role in CD, whilst the Th-2 response may be involved in UC (12,13). IL-5 and IL-13 are characteristic Th-2 cytokines involved in UC (12). It has been previously demonstrated that the overexpression of IL-5 significantly increases the severity of OXA-induced colitis. Lack of IL-5 has been shown to attenuate the accumulation of eosinophils in the colon of dextran-sodium sulfate-treated mice (14). Eosinophils may reduce the barrier properties of epithelial cell monolayers. In addition, preliminary evidence suggests that eosinophil activation results in an increase in epithelial permeability in biopsy specimens from patients with UC (15). Eosinophils may enhance intestinal inflammation via the induction of a reduction in epithelial barrier function (16).

In the present study, no adverse effects of Banxiaxiexin decoction were observed. However, the safety of this decoction is not yet known, although it is widely believed that it is safe to use herbal medicines for the treatment of various diseases in China. Since numerous adverse effects of Chinese herbal medicines have been reported, the long-term safety of Banxiaxiexin decoction requires further investigation.

## Figures and Tables

**Table I:** Disease activity index.

Score	Percentage weight loss	Stool consistency	Hematochezia level
0	0	Normal	Negative hemoccult
1	1–5		
2	5–10	Mushy	Positive hemoccult
3	10–15		
4	≥15	Diarrhea	Blood traces visible in stool

**Table II:** Scoring system for inflammation-associated histological changes in the colon.

Score	Histological changes
0	No evidence of inflammation
1	Low level of inflammation with scattered infiltrating mononuclear cells (1–2 foci)
2	Moderate inflammation with multiple foci
3	Severe inflammation with increased vascular density and marked wall thickening
4	Maximal severity of inflammation with transmural leukocyte infiltration and loss of goblet cells

**Table III:** Scores for inflammation-associated histological changes (mean ± SD).

Parameter	Model	SASP	Banxia	Control
N	10	10	10	10
Scores	2.7±0.7	1.3±0.6	1.5±0.4	1.7±0.5

SASP, sulfasalazine; SD, standard deviation.

## Abbreviations

IBD: inflammatory bowel diseases
UC: ulcerative colitis
CD: Crohn’s disease
5-ASA: 5-aminosalicylic acid
CAM: complementary and alternative medicine
OXA: oxazolone
SASP: sulfasalazine
DAI: disease activity index
DSS: dextran-sodium sulfate
